# Hypomethylating Agent Azacitidine Is Effective in Treating Brain Metastasis Triple-Negative Breast Cancer Through Regulation of DNA Methylation of Keratin 18 Gene

**DOI:** 10.1016/j.tranon.2020.100775

**Published:** 2020-05-11

**Authors:** Christopher Butler, Samuel Sprowls, Gabor Szalai, Tasneem Arsiwala, Pushkar Saralkar, Benjamin Straight, Shea Hatcher, Evan Tyree, Michael Yost, William J. Kohler, Benjamin Wolff, Emily Putnam, Paul Lockman, Tuoen Liu

**Affiliations:** a Department of Biomedical Sciences, West Virginia School of Osteopathic Medicine, 400 Lee Street North, Lewisburg, WV; b Department of Pharmaceutical Sciences, College of Pharmacy, West Virginia University, Morgantown, WV; c Department of Biomedical Sciences, Burrell College of Osteopathic Medicine, Las Cruces, NM; d Zymo Research Corporation, Irvine, CA

## Abstract

Breast cancer patients presenting with symptomatic brain metastases have poor prognosis, and current chemotherapeutic agents are largely ineffective. In this study, we evaluated the hypomethylating agent azacitidine (AZA) for its potential as a novel therapeutic in preclinical models of brain metastasis of breast cancer. We used the parental triple-negative breast cancer MDA-MB-231 (231) cells and their brain colonizing counterpart (231Br) to ascertain phenotypic differences in response to AZA. We observed that 231Br cells have higher metastatic potential compared to 231 cells. With regard to therapeutic value, the AZA IC_50_ value in 231Br cells is significantly lower than that in parental cells (*P* < .01). AZA treatment increased apoptosis and inhibited the Wnt signaling transduction pathway, angiogenesis, and cell metastatic capacity to a significantly higher extent in the 231Br line. AZA treatment in mice with experimental brain metastases significantly reduced tumor burden (*P* = .0112) and increased survival (*P* = .0026) compared to vehicle. Lastly, we observed a decreased expression of keratin 18 (an epithelial maker) in 231Br cells due to hypermethylation, elucidating a potential mechanism of action of AZA in treating brain metastases from breast cancer.

## Introduction

Breast cancer is the second leading cause of cancer with the highest mortality rate in females in the United States [1]. Based on genetic profiling, breast cancer is classified into four subtypes: luminal A (ER+ and/or PR+, HER2−, low Ki67 index), luminal B (ER+ and/or PR+, HER2+ or −, high Ki67 index), HER2 positive (HER2+, ER−, PR−), and triple negative (ER−, PR−, and HER2−) [2]. For drug treatment of receptor-positive breast cancers, therapeutics that specifically target the hormone receptors and HER2 are available [3]. However, for triple-negative breast cancer, which is associated with an unfavorable prognosis, there are no targeted therapies, leaving patients with traditional chemotherapeutic agents that have significant adverse effects [4]. In some patients, tumors metastasize to different locations within the body including lungs, liver, bones, or brain. Once the lesion disseminates to brain, average patient survival time is less than 1 year, and treatments including chemotherapy, radiation, and surgery are the primarily palliative options [5].

DNA methylation is an epigenetic mechanism used by cells to control gene expression [6]. In normal cells, DNA methylation ensures proper regulation of gene expression and silencing. Abnormal DNA hypermethylation may result in dysregulation of these mechanisms, resulting in altered gene function [7]. Cancer is associated with altered DNA methylation leading to inhibition of tumor suppressor genes and compaction of chromatin [8,9]. Hypermethylation of numerous tumor suppressor genes is recognized in multiple cancer types, and this phenomenon may contribute to the initiation and/or progression of the disease [10,11]. Of relevance to this study, multiple genes that are critical in breast carcinogenesis are hypermethylated including the tumor suppressors p16, p53, and BRCA1; cell cycle regulator CCND2; and cell growth regulators ER and PR, as well as others [[12], [13], [14]].

Hypomethylating agents such as azacitidine (or azacytidine, AZA) and its deoxyl-derivative decitabine have been approved by the US FDA to treat patients with hematological malignancies such myelodysplastic syndromes (MDS) [15]. Although their use in breast cancer treatment is not approved for clinical use, DNA hypomethylating agents have been shown to activate tumor suppressor genes. It should be noted that when given intravenously, AZA crosses the blood–brain barrier (BBB) and reaches cytotoxic levels in cerebrospinal fluid [16,17]. In this study, we evaluated the antitumor effects of the hypomethlyating agent AZA in a preclinical model of triple-negative breast cancer brain metastasis and observed that AZA has efficacy as a novel chemotherapeutic agent.

## Materials and Methods

### Cell Lines and Chemicals

The parental regular triple-negative breast cancer MDA-MB-231 (abbreviated as “231”) cell line was purchased from ATCC (Manassas, VA). The brain colonizing counterpart of 231 cells (“231Br” cells), also known as tropic or brain-seeking 231 Br cells, were isolated from brain lesions in the brain metastasis of breast cancer mouse model we previously generated. These cells were kindly provided by Dr. Patricia Steeg from the National Institute of Health Center for Cancer Research. Both cell lines were cultured at 37°C, 5% CO_2_, in Dulbecco's modification of Eagle's medium (Corning, Inc., Corning, NY) containing 10% fetal bovine serum (VitaScientific, Inc., College Park, MD), 10 mM L-glutamine (Thermo Fisher Scientific, Waltham, MA), and 1× penicillin/streptomycin (Thermo Fisher Scientific, Waltham, MA). AZA was purchased from Sigma-Aldrich Inc. (St. Louis, MO).

### Western Blotting

The Western blotting assay was described previously [18]. Briefly, cell lysates were prepared in RIPA buffer, and protein samples were loaded on a SDS-polyacrylamide gel, separated by electrophoresis, and subsequently transferred to a PVDF membrane. Membranes were blocked with 5% milk in 1× TBS containing 0.05% (v/v) Tween-20 for 4 hours at room temperature and washed seven times with 1× TBS and 1× TBST alternatively. Membranes were then incubated with primary antibody overnight at 4°C followed by incubation with secondary antibody at room temperature for 1 hour. Pierce supersignal chemiluminescent substrates were used, and images were captured by using the G:BOX Chemi XX9 gel doc system (Syngene Inc., Frederick, MD). Detailed information of the antibodies is listed in Supplementary Table 1.

### MTT Assay

The cell viability was measured using the MTT assay kit (ATCC, Inc., Manassas, VA), and the manufacturer’s protocol was followed. Briefly, 1000 cells in 100 μl were plated in each well in a 96-well plate and incubated overnight. On the next day, the cell medium was replenished, and various concentrations of AZA were added to each well (triplicate) accordingly and incubated at 37°C for 72 hours. After incubation, 10 μl of MTT reagent was added to each well, and the plate was incubated at 37°C for 4 hours. A total of 1000 μl of detergent reagent was then added to each well, and the plate was left at room temperature in the dark for 4 hours. The optical density of absorbance at 570 nm was recorded using a Synergy2 multimode microplate reader (Biotek, Inc., Winooski, VT). The cell viability was calculated based on the optical density value normalized to blank control. The IC_50_ of AZA in 231 and 231 Br cells was calculated based on the cell viability measured by three independent MTT assays.

### Apoptosis Assay

Cell apoptosis was measured using the PE Annexin V apoptosis detection kit (BD Biosciences, Inc., San Jose, CA). The manufacturer's protocol was followed, and the percentage of apoptotic cells was detected and analyzed using the BD Accuri C6 flow cytometry (BD Biosciences, Inc., San Jose, CA).

### Enzyme-Linked Immunosorbent Assay (ELISA)

After cells were treated with various concentrations of AZA for 72 hours, the secreted vascular endothelial growth factor (VEGF) in the medium was measured by using the human VEGF ELISA kit (Sigma-Aldrich, Inc., Saint Louis, MO). The ELISA was described previously, and the manufacturer's protocol was followed [19]. Briefly, 100 μl of each standard and medium sample was mixed and added into 96-well plates and incubated for 2.5 hours at room temperature with gentle shaking. The supernatant was then discarded and washed four times with 1× washing solution. A total of 100 μl of 1× prepared biotinylated detection antibody was added for 1 hour at room temperature with gentle shaking. The solution was discarded and washed, and 100 μl of prepared HRP-conjugated streptavidin solution was added and incubated for 45 minutes at room temperature with gentle shaking. The solution was discarded, and 100 μl of ELISA colorimetric TMB reagent was added and incubated for 10 minutes at room temperature in the dark with gentle shaking. Finally, 50 μl of stop solution was added, and the plate was read at 450 nm using a Synergy2 multimode microplate reader (Biotek, Inc., Winooski, VT). The amount of VEGF present in the cell culture medium was normalized to the number of cells present at the time of collection.

### *In Vitro* Cell Migration Assay

The Transwell migration assay was described previously [20,21]. After cells were treated with various concentration of AZA for 72 hours, they were washed with PBS and resuspended in serum-free medium. Six hundred microliters of regular medium containing 10% serum was added to one well of a 24-well plate, and then the migration chamber (Millipore Inc., PI8P01250) was replaced in the well. One hundred microliters of serum-free medium was first added in each chamber, and then a total of 10^5^ cells in 200 μl serum-free medium was added to the chamber. The plates were incubated at 37°C for various times (3, 16, and 72 hours). At the end of the designated time point, medium in the chamber was removed, and the chambers were gently washed twice with PBS. Cells were fixed with formaldehyde (3.7% in PBS) at room temperature for 20 minutes followed by PBS wash and permeabilization by 100% methanol at room temperature for 20 minutes. After removal of methanol and washing with PBS, cells were stained with 1% crystal violet at room temperature for 20 minutes. Excess crystal violet was removed, and cells were washed with PBS. Finally, cells on the chamber were counted under the light microscope (average number of five microscope fields).

### *In Vitro* Cell Invasion Assay

The cell invasion assay was described previously [20,21]. Twenty-four-well plates containing Matrigel invasion chambers (Corning Inc., Corning, NY) were preincubated at 37°C overnight. Similar to the procedure used in the cell migration assay, the same number of cells (10^5^ cells in 200 μl serum-free medium) was plated in each well, and the plates were incubated at 37°C for predesignated periods (16, 72, and 96 hours). After reaching the time point, cells were fixed, permeabilized, stained, and counted under the light microscope using the same techniques as the cell migration assay.

### Wound-Healing Assay

The wound-healing assay (also known as *in vitro* scratch assay) has been described previously [20,21]. A total of 10^6^ of the 231 and 231Br cells were plated in six-well plates and incubated at 37°C overnight. On the next day, after confirming that the cells were attached to the well and cell confluence reached ~70%, a scratch was made in each well using a 1-ml pipette tip, and medium containing increasing concentrations of AZA was added to each replicate. The number of cells present in the scratch made on day 0 was counted for each predesignated time (24, 48, 72, and 96 hours), and pictures of the denuded area were taken using an Olympus IX50 inverted system microscope (Olympus, Inc., Center Valley, PA) every day for 5 days.

### Detection of the Keratin 18 Gene by Polymerase Chain Reaction (PCR)

DNA from both cell lines was extracted and purified using the GeneJet genomic DNA purification kit (Thermo Fisher Scientific, Waltham, MA) based on the manufacturer's protocol. The pair of primers designed to measure the keratin 18 gene by PCR is forward 5′-CTGGCCTCTTACCTGGACAGAGTGAG-3′and reverse 5′-TGT GGCTAGGTGCGCGGATGGAAATCC-3′, which yields a 300-bp PCR product. The PCR was set up by using the iProof high-fidelity PCR kit (Bio-Rad Laboratories, Inc., Hercules, CA) and was performed with an Eppendorf Mastercycler thermocycler (Hamburg, Germany). The PCR thermal cycling protocol was as follow: initial denaturation at 98°C for 30 seconds, denaturation at 98°C for 10 seconds, annealing at 65°C for 30 seconds, and extension at 72°C for 30 seconds, a total of 30 cycles, and final extension at 72°C for 10 minutes.

### Real-Time PCR

The real-time PCR procedure was described previously [18]. Briefly, cells were harvested by centrifugation at 1500*g* for 5 minutes at 4°C, resuspended in 250 μl 1× PBS, and then lysed by adding 750 μl Trizol LS reagent (Invitrogen, Inc., Carlsbad, CA). RNA was then isolated following the manufacturer's protocol and was subsequently resuspended in 30 μl of RNase-free water. The RNA concentration was measured using a Synergy2 multimode microplate reader (BioTek Inc., Winooski, VT). The TURBO DNA-free kit (Thermo Fisher Scientific Inc., Waltham, MA) was used to remove DNA contamination within each sample. The first-strand cDNA was synthesized using the SuperScript III first-strand synthesis system (Thermo Fisher Scientific Inc., Waltham, MA) following the manufacturer's protocol. FAM-MGB primer/probe mixes for keratin 18 (Hs02827483_g1), VEGFA (Hs00900055_m1), and GAPDH (Hs02786624_g1) were used for real-time PCR TaqMan gene expression assays (Applied Biosystems Inc., Foster City, CA). All real-time PCRs were performed in duplicate with no-RT control and water control on the StepOnePlus real time PCR system (Applied Biosystems Inc., Foster City, CA). Individual cDNA samples were normalized according to their levels of GAPDH, and the relative standard curve method was used for analysis.

### Sequencing of the Intron 1 Region of the Keratin 18 Gene in Both Cell Lines

In order to compare the DNA sequence of intron 1 region (737 bp) of the keratin 18 gene between 231 and 231 Br cells, we designed a pair of primers and used PCR to amplify the desired region. The forward sequence was 5′-GATCATCGAGGACCTGAGGG-3′; the reverse sequence was 5′-GGGGAGC AGATCCTTCTTAGC-3′. The PCR was set up using the DreamTaq hot start green DNA polymerase kit (ThermoFisher Scientific, Inc., Waltham, MA), and PCR was performed with the Bio-Rad MJ mini personal thermal cycler # PTC114 (Bio-Rad Laboratories, Inc., Hercules, CA). The PCR thermal cycling protocol was as follows: initial denaturation at 95°C for 2 minutes, denaturation at 95°C for 30 seconds, annealing at 60°C for 30 seconds, and extension at 72°C for 45 seconds, a total of 19 cycles, then followed by dropping 0.5°C each time to 50°C, 95°C for 30 seconds, 50°C for 30 seconds, 72°C for 30 seconds, a total of 19 times. The final extension was at 72°C for 10 minutes. This yielded a single and clear 906-bp PCR product. The PCR product was cloned into the pCR2.1-TOPO vector using the TOPO TA cloning kit (Invitrogen, Inc., Carlsbad, CA) following the manufacturer's protocol. The cloning product was then transformed into DH5-alpha *E. coli* competent cells (Invitrogen, Inc., Carlsbad, CA). The transformations were spread on ampicillin-selective plates and incubated overnight at 37°C. Colonies were picked and cultured in LB medium containing 100 μg/ml ampicillin with shaking at 250 rpm overnight at 37°C. On the next day, plasmid DNA was isolated by using the Invitrogen PureLink quick plasmid miniprep kit (Invitrogen, Inc., Carlsbad, CA). Plasmid DNA samples from five positive colonies were sent to the West Virginia University Genomics Core Facility for sequencing.

### Keratin 18 Gene DNA Methylation Determination by Bisulfite Chemical Modification

The genomic DNA from 231 and 231Br cells was isolated using the GeneJET genomic DNA purification kit (ThermoFisher Scientific, Inc., Waltham, MA) following the manufacturer's protocol. The genomic DNA was then treated with bisulfite to chemically modify nonmethylated cytosines into uracil using the EZ DNA methylation-lightning kit (Zymo Research, Inc., Irvine, CA) following the manufacturer's protocol. In this treatment, unmethylated cytosine residues were converted to uracil, while methylated cytosine residues were resistant to bisulfite modification and remained as a cytosine residue. In order to measure and compare the DNA methylation of the keratin 18 gene in both cell lines, five pairs of primers were designed and used to fully cover and amplify the bisulfite modified intron 1 region of keratin 18 gene by PCR. The sequences of the five pairs of primers were as follows: pair 1 forward: 5′-TTAATTATYGGTTTTTG GGTTTTGTTTAGG-3′, reverse: 5′-RATCTCCAAACTCCTCA CTCTAT-3′; pair 2 forward: 5′-TTGGATAGAGTGAGGAGTTTGGAGA-3′, reverse: 5′-AAAAATCCAAATATACCC AACCCCCT-3′; pair 3 forward: 5′-GGAGGGGGTTGGGTATATTT-3′, reverse: 5′-CACCC TAAATTAACTCCTCCCAAAA-3′; pair 4 forward: 5′-TTGAGTTATTTAGGAGTAAAT AAGAGGTTTTTTTTTG-3′, reverse: 5′-CCAAAAATAACCAAAAACTCTCCCTAAA-3′; pair 5 forward: 5′-TGGTTATTTTTGGGATTAGGAAGTTTTTATTAG-3′, reverse: 5′-CAAA ATCCCACTATAAACCCCTAACT-3′. The methods used in the PCR setup and performance, TOPO TA cloning, and plasmid DNA isolation were the same as described above. Each of the five pairs of primers yielded a single and clear PCR band using bisulfite converted genomic DNA as the template from both cell lines (Supplementary Figure 4). Finally, plasmid DNA samples from five positive colonies generated from each pair of primers were sent to West Virginia University Genomics Core Facility for sequencing.

### HhaI Restriction Digestion

The HhaI restriction enzyme was purchased from New England BioLabs, Inc. (Ipswich, MA). A 20-μl restriction digestion reaction containing 500 ng genomic DNA isolated from 231 or 231Br cells and 1 μl HhaI was set up following the manufacturer's protocol. The reaction was incubated in a 37°C water bath overnight allowing the full digestion of DNA by HhaI. The pair of primers designed used to detect the HhaI digestion site is forward: 5′-GGAGGGGGTTGGG CATACT-3′, reverse: 5′-CACCCTGGATTGGCTCCTCCCAAAG-3′. If DNA methylation prevented digestion of the keratin 18 gene, the DNA would not be digested by HhaI, and this primer would yield a ~300-bp PCR product. On the contrary, if the DNA was digested by HhaI, no such ~300-bp PCR product would be formed. A pair of primers designed and used as positive control to detect the keratin 18 gene was forward: 5′-AGCTAGAC AAGTACTGGTCTCAGCAG-3′, reverse 5′-CAGCTCTGACTCAAGGTGCAGCAGGAT-3′. Regardless of digestion status of the keratin 18 gene digested by HhaI, this primer could detect the presence of the keratin 18 yielding a ~300-bp PCR product in both cell lines. Methods used in PCR setup and performance were the same as described above.

### Survival of Animals with Preclinical Brain Metastases of Breast Cancer Treated with AZA

Animal experiments were approved by the Institutional Animal Care and Use Committee at the West Virginia University. Use of the brain metastasis breast cancer *in vivo* mouse model has been described previously [22,23]. Briefly, the brain tropic breast cancer 231Br cells were isolated by repeated cycles of intracardiac injection of the parental 231 cells, harvesting of brain metastases, and *ex vivo* culture of isolated cells. These brain metastatic cells were injected into the left cardiac ventricle; were circulated in the peripheral vasculature; were arrested in brain capillaries, with subsequent extravasation across the *in vivo* BBB; and developed metastatic lesions in mice. The presence of metastatic tumors was confirmed on day 21 after intracardiac injection with bioluminescent imaging (BLI) using the IVIS Spectrum CT imaging system (PerkinElmer, Waltham, MA). After tumor burden confirmation on day 21, mice were intraperitoneally administered with AZA (2.5 mg/kg) or vehicle control (PBS) in a total of four cycles. In each cycle, AZA or PBS was injected for 5 continuous days, stopped for 2 days, and then again followed by another 5 continuous days. Between each cycle, there was a 2-week off interval. Tumor burden was monitored twice weekly and quantified using BLI assay, similar to our previous work [22,23]. Mice were introperitoneally injected with D-luciferin potassium salt (150 mg/kg body weight, PerkinElmer Inc., Waltham, MA), and then the brain bioluminescent signal was captured 15 minutes after injection of luciferin. Animals were euthanized under anesthesia at the presentation of neurological symptoms or when moribund.

### Statistics

Statistical significance of the data between two groups was analyzed by the Student’s *t* test (Prism 8). Statistical significance of the data with more than two groups was analyzed by one-way ANOVA with a Tukey posttest (Prism 8). Significance levels were set at *P* < .05 (*), *P* < .01 (**), and *P* < .001 (***).

## Results

### Brain Colonizing Breast Cancer Cells Display a Different Growth Pattern Compared to Parental Breast Cancer Cells

**Figure 1:**
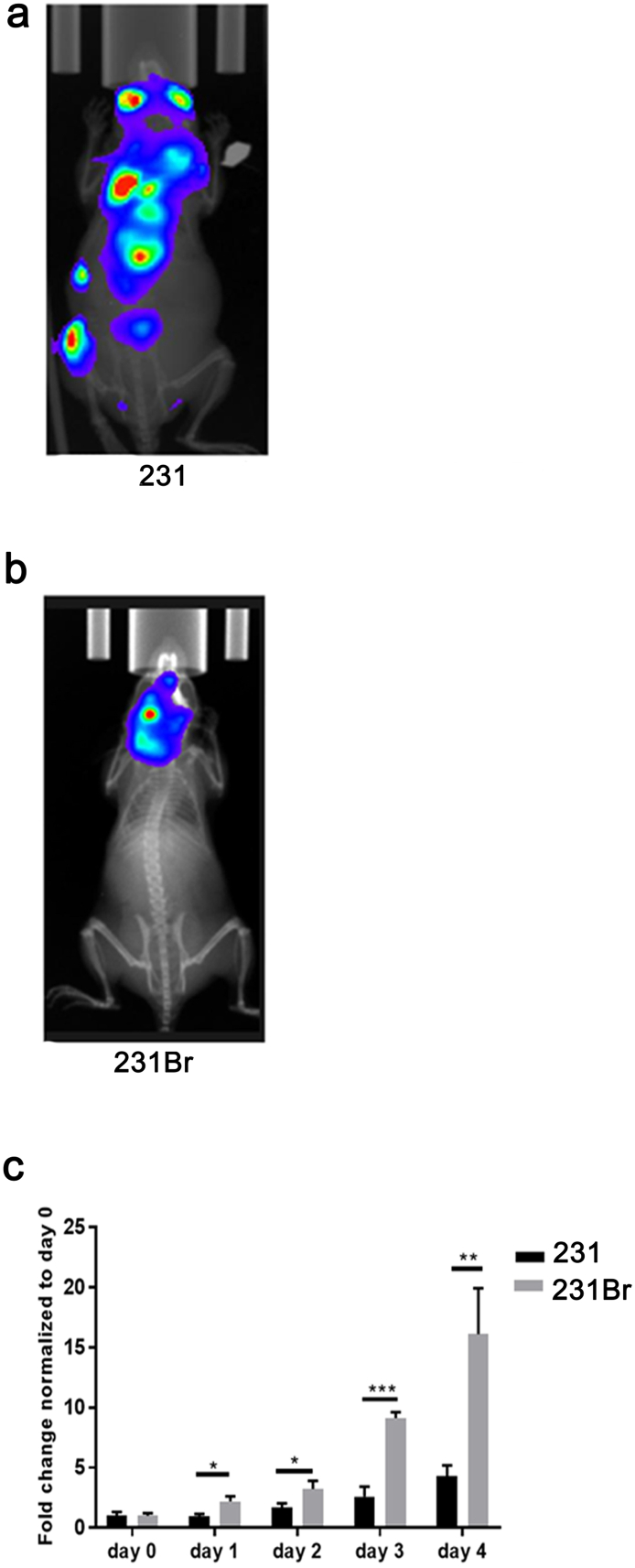
Brain colonizing breast cancer cells (231Br) have a different oncological phenotype compared to parental breast cancer 231 cells. (A) After intracardiac injection, the parental triple-negative breast cancer cells (231) are seen disseminated throughout the body of mice. (B) The brain colonizing triple-negative breast cancer cells (231Br) primarily reside in the brain of mice. (C) 231Br cells grow significantly faster compared to 231 cells *in vitro*. Note: images acquired for (A) and (B) are representative images taken on day 30 following intracardiac injection of either 231 or 231Br cells. Fold change of cell numbers in each day was compared between the two cell lines using Student's *t* test. All error bars represent standard deviation (SD), *N* = 3 technical replicates, representative of two independent experiments. *P < .05, **P < .01, ***P < .001.

After intracardiac injection, the parental triple-negative breast cancer cells (231) are disseminated throughout the body (Figure 1*A*), while the brain colonizing cells (231Br) are primarily distributed to the brain (Figure 1*B*). The fold change of cell growth suggested that the 231Br cells replicate faster than the 231 cells (Figure 1*C*).

### Brain Colonizing Breast Cancer Cells Are More Sensitive to AZA Treatment Compared to Regular Cancer Cells

**Figure 2:**
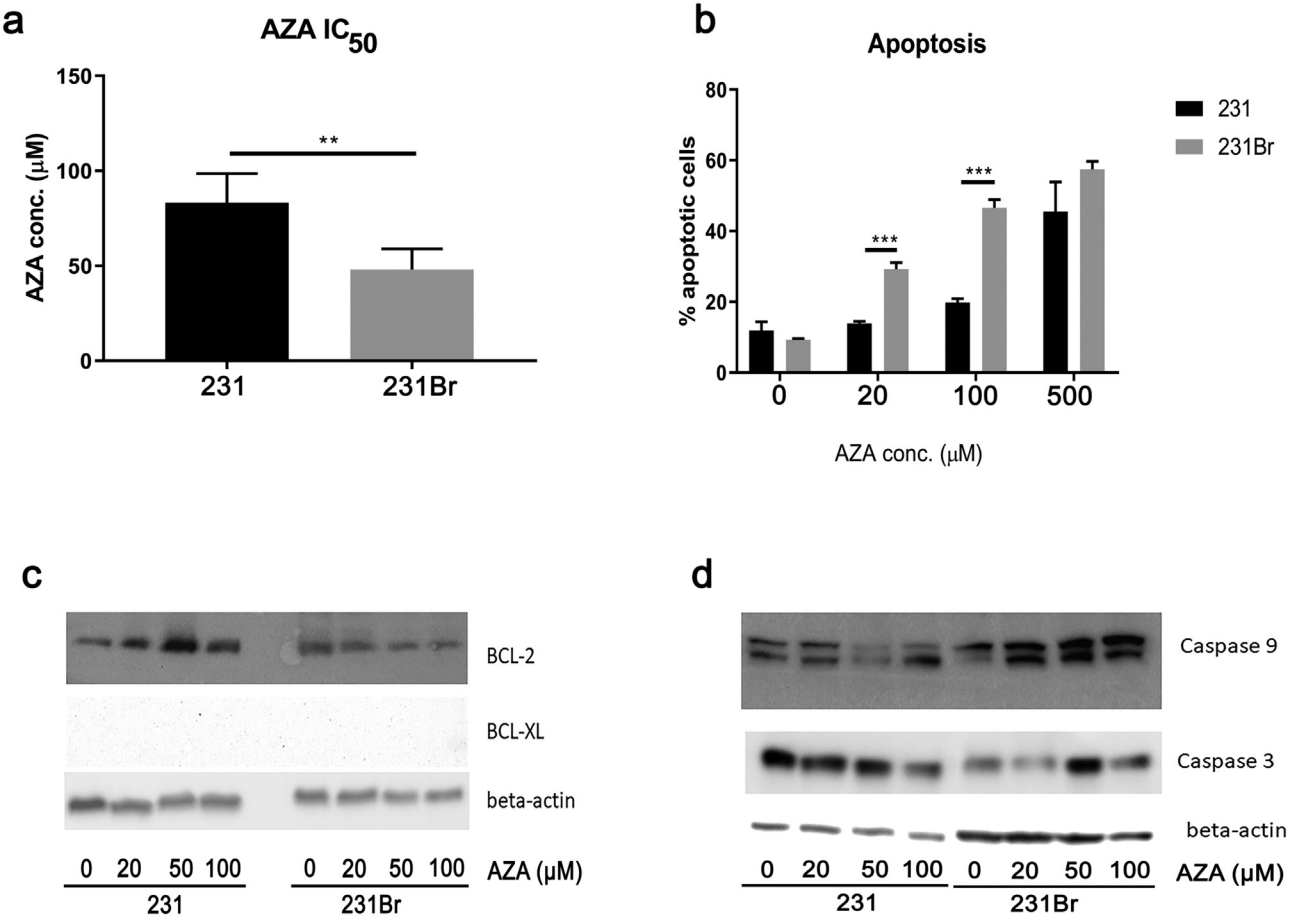
231Br breast cancer cells are more sensitive to AZA treatment compared to 231 cells. (A) IC_50_ values of AZA in both cell lines were calculated using the MTT assay. The IC_50_ value of AZA is 48 ± 4.90 μM in 231Br cells and 83.33 ± 8.82 μM in 231 cells (*P* < .01). (B) 231 and 231Br cells were treated with various concentrations of AZA for 72 hours, and the Annexin V–positive cells were considered as apoptotic cells. Treatment with 20 μM and 100 μM of AZA triggers higher percentage of apoptotic cells in 231Br cells compared to 231 cells. IC_50_ values and percentage of Annexin V–positive cells were compared between the two cell lines using Student's *t* test. All error bars represent SD, *N* = 3 technical replicates, representative of three independent experiments. *P < .05, **P < .01, ***P < .001. (C) Expression of BCL-2 and BCL-xL in 231 and 231Br cells after AZA treatment for 72 hours measured by Western blotting assay. Beta-actin was used as the loading control. The blots shown are a presentation of two independent experiments. (D) Expression of caspase-3 and caspase-9 in 231 and 231Br cells after AZA treatment for 72 hours. Beta-actin was used as the loading control. The blots shown are a presentation of two independent experiments.

The IC_50_ value of AZA in 231Br and 231 cells was determined using an MTT assay. We observed IC_50_ values for AZA of 83.3 ± 8.8 μM in 231Br cells and 48 ± 4.9 μM in 231 cells (*P* < .01, Figure 2*A* and Supplementary Figure 1*A*), suggesting differential sensitivity to AZA. In subsequent experiments, both lines were treated with a range of AZA concentrations (0-500 μM) for 72 hours, and apoptosis was calculated by a percentage of Annexin V–positive cells using flow cytometry. At concentrations of 20 μM and 100 μM of AZA, a greater degree of apoptosis was induced in 231Br cells compared to 231 cells (*P* < .001). However, 500 μM of AZA caused apoptosis at similar level in both cell types (Figure 2*B* and Supplementary Figure 1*B*). The expression of the antiapoptotic protein BCL-2 decreased in 231Br cells upon AZA treatment, with little effect in the 231 cells. However, expression BCL-xL was not detected in either cell line (Figure 2*C* and Supplementary Table 2). Further, there was an increased expression of the proapoptotic proteins caspase-3 and caspase-9 when AZA was exposed to the 231Br cells in a dose-dependent manner (Figure 2*D* and Supplementary Table 2). However, expression of two other proapoptotic proteins, BAD and BAX proteins, remained unchanged after AZA treatment (Supplementary Figure 1*C*).

### AZA Inhibited the Wnt Signaling Transduction Pathway in Brain Colonizing Breast Cancer Cells

**Figure 3:**
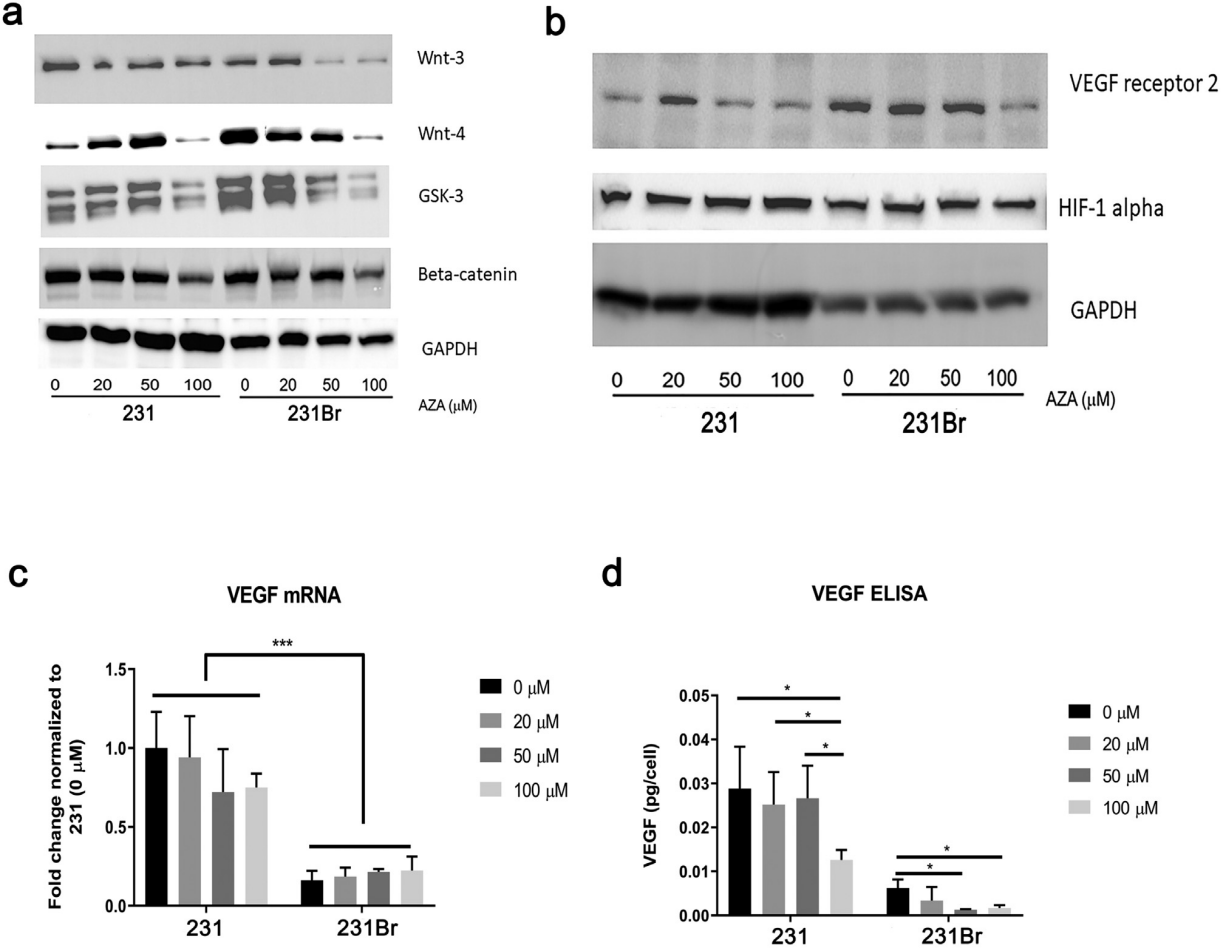
AZA differentially inhibits Wnt signaling transduction pathway and angiogenesis-related markers *in vitro*. (A) Expression of Wnt-3, Wnt-4, GSK-4, and beta-catenin in 231 and 231Br cells after AZA treatment for 72 hours measured by Western blotting assay. GAPDH was used as the loading control. The blots shown are a presentation of two independent experiments. (B) Expression of VEGF receptor 2 and hypoxia-inducible factor-1 alpha in 231 and 231Br cells after AZA treatment for 72 hours measured by Western blotting assay. GAPDH was used as the loading control. The blots shown are a presentation of two independent experiments. (C) VEGF mRNA level in 231 and 231Br cells after AZA treatment for 72 hours measured by real-time PCR. All error bars represent SD, *N* = 3 technical replicates, representative of two independent experiments. *P < .05, **P < .01, ***P < .001. (D) The amount of VEGF released into the cell culture medium of 231 and 231Br cells after 72 hours of AZA treatment measured by ELISA. All error bars represent SD, *N* = 3 technical replicates, representative of two independent experiments. *P < .05, **P < .01, ***P < .001.

Treatment with AZA in 231Br cells for 72 hours inhibited expression of Wnt-3, Wnt-4, glycogen synthase kinase-3 (GSK-3), and beta-catenin in a dose-dependent manner as determined by a Western blot (Figure 3*A* and Supplementary Table 2). In contrast, treatment with AZA in 231 cells did not change Wnt-3 expression significantly, but at a higher concentration (100 μM), AZA inhibited expression of Wnt-4, GSK-3, and beta-catenin in these cells (Figure 3*A* and Supplementary Table 2). AZA treatment had no effect on Wnt-1 expression in either cell types and the expression of Wnt-5, Wnt-11, and adenomatous polyposis coli was undetectable in both lines (Supplementary Figure 2*A*). While there were changes in the Wnt signaling transduction pathway, there was no significant impact on either the Ras/Raf/MEK/MAPK or the PI3K/Akt/mTOR pathways in either cell line (Supplementary Figure 2, *B* and *C*).

### Angiogenesis-Related Markers Were Decreased by AZA Treatment in Both Cell Lines

Angiogenesis-related markers were measured in both cell lines treated by different concentrations of AZA. At higher concentrations (100 μM), AZA treatment for 72 hours decreased expression of VEGF receptor 2 only in the 231Br cells. Treatment with AZA had no significant impact on hypoxia-inducible factor-1 alpha expression in either cell line (Figure 3*B*). However, expression of VEGF, VEGF receptor 1, and transforming growth factor beta was undetectable in either type of cells (data not shown). The mRNA was measured by real-time PCR (Figure 3*C*) and the secreted VEGF into the cell culture medium was measured by ELISA assay (Figure 3*D*) in both lines treated with AZA. Cellular VEGF mRNA level was significantly higher in 231 cells compared to 231Br cells (*P* < .001), and AZA treatment did not have a significant impact on VEGF mRNA levels in both cell lines (Figure 3*C*). However, AZA reduced the amount of VEGF secreted into the medium in a dose-dependent manner in both cell lines (Figure 3*D*).

### Brain Colonizing Cells Have Higher Migration and Invasion Potential

**Figure 4:**
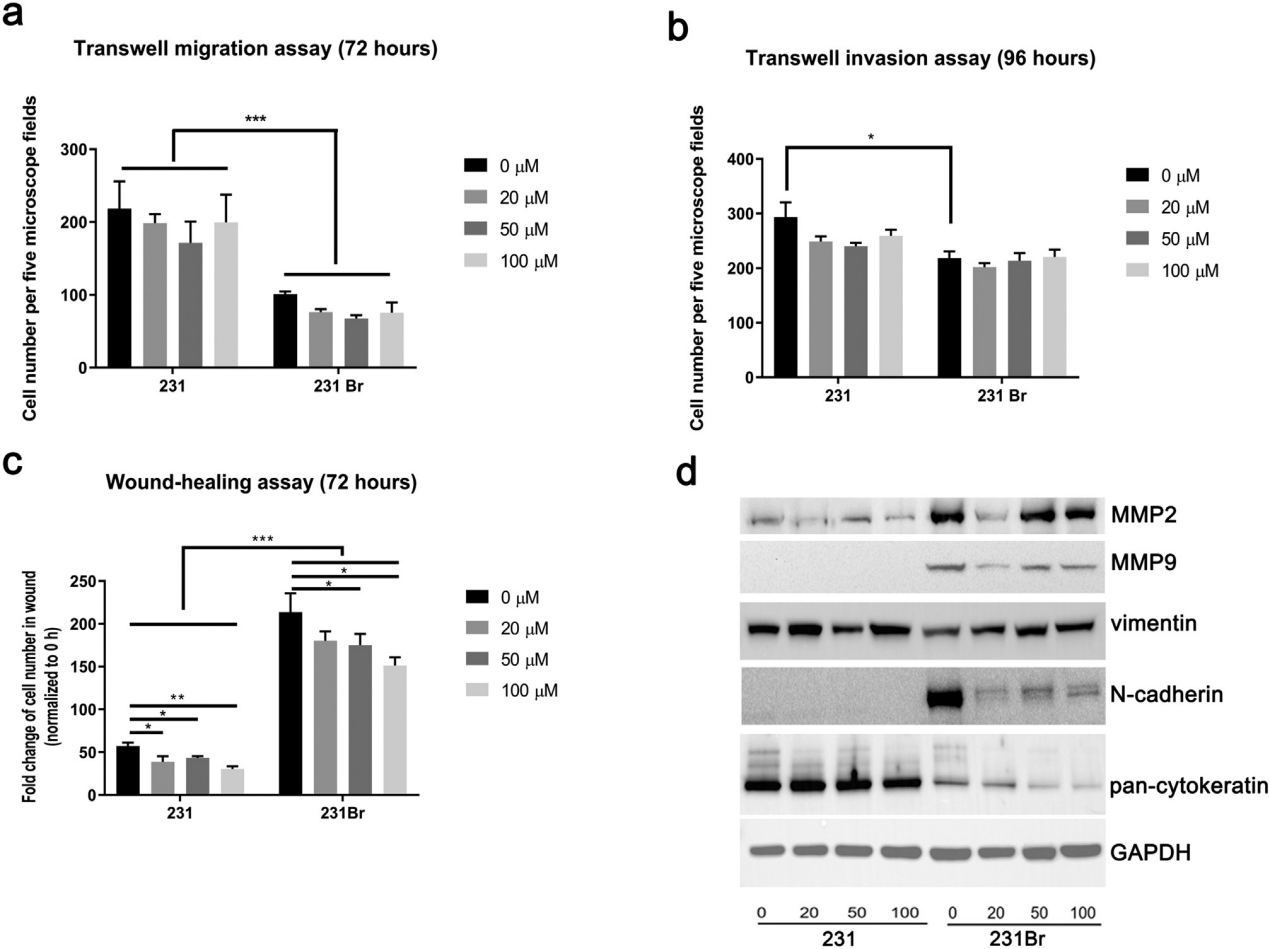
231Br cells have higher migration and invasion potential compared to 231 cells. (A) Quantification of cells migrating across Transwells 72 hours after plating cells in the migration chambers measured by Transwell migration assay. *Y*-axis stands for the average number of cell migration per five microscope fields. All error bars represent SD, representative of two independent experiments. *P < .05, **P < .01, ***P < .001. (B) Quantification of cells migrating across Transwells 96 hours after plating cells in the Matrigel-coated migration chambers measured by Transwell invasion assay. *Y*-axis stands for the average number of cell migration per five microscope fields. All error bars represent SD, representative of two independent experiments. *P < .05, **P < .01, ***P < .001. (C) Quantification of cells presented in the scratch made on day 0 (0-hour time point) at 72 hours after AZA treatment by wound-healing assay. Cell numbers in the scratch wound were normalized to 0 hour. All error bars represent SD, representative of two independent experiments. *P < .05, **P < .01, ***P < .001. (D) Expression of MMP2, MMP9, vimentin, N-cadherin, and pan-cytokeratin in 231 and 231Br cells after AZA treatment for 72 hours measured by Western blotting assay. GAPDH was used as the loading control. The blots shown are a presentation of two independent experiments.

After incubation, we did not observe significant changes in cell number with AZA at early time points, but after 72 hours of incubation, 231Br cells had higher migration potential (*P* < .001, Figure 4*A* and Supplementary Figure 3*A*). Similarly, in the cell invasion assay, at early time points (16 and 72 hours), there were no significant differences between the cell lines (Supplementary Figure 3*B*), but at 96 hours, 231Br cells (without AZA) had increased migration (*P* < .05, Figure 4*B*). Of interest, AZA treatment did not significantly impact cell invasion in either line (Figure 4*B* and Supplementary Figure 3*B*). Consistent with cell migration and invasion assays, the wound-healing assay showed that the 231Br cells migrated faster than 231 cells. After 72 hours of AZA treatment, the width of wound was still present in 231 cells but not in 231Br cells (Supplementary Figure 3, *D*-*E*). In addition, AZA treatment significantly inhibited wound healing in both lines after 48 hours (Figure 4*C* and Supplementary Figure 3, *C*-*E*).

### Hallmark Metastasis Markers Are Higher in the Brain Colonizing Cell Line

Matrix metalloproteinases (MMPs) such as MMP2, MMP7, and MMP9 are metastasis markers in breast cancer [24,25]. MMP2 was expressed in both cell lines but was greater in 231Br cells, and MMP9 expression was not seen in 231 cells but was detected in 231Br cells. Interestingly, high concentrations of AZA (50 and 100 μM) treatment for 72 hours had no significant impact on expression of MMP2 and MMP9 in either cell line (Figure 4*D* and Supplementary Table 2). Epithelial markers including E-cadherin and cytokeratins, and mesenchymal marker N-cadherin were measured with and without AZA treatment in both cell lines [26]. Expression of E-cadherin was not detected in either cell line (data not shown), while vimentin was present at similar levels in both cell types. N-cadherin expression was only detected in 231Br cells. Treatment with AZA decreased expression of N-cadherin in 231 cells (Figure 4*D* and Supplementary Table 2). Since cytokeratin expression is decreased during the EMT process [27], we measured their expression profile using a pan-cytokeratin antibody mixture of AE1 and AE3. This was done to detect multiple members of the cytokeratin family of proteins [[1], [2], [3], [4], [5], [6], [7], [8], [9], [10],[14], [15], [16],^19^,and]. We observed that expression of cytokeratins measured by the pan-cytokeratin antibody was lower in 231Br cells compared to 231 cells. In addition, higher concentrations of AZA (50 μM and 100 μM) treatment further decreased expression of pan-cytokeratin in the 231Br line (Figure 4*D* and Supplementary Table 2).

### DNA Methylation Is Altered in Brain Colonizing Cells Compared to Parental Cancer Cells

**Figure 5:**
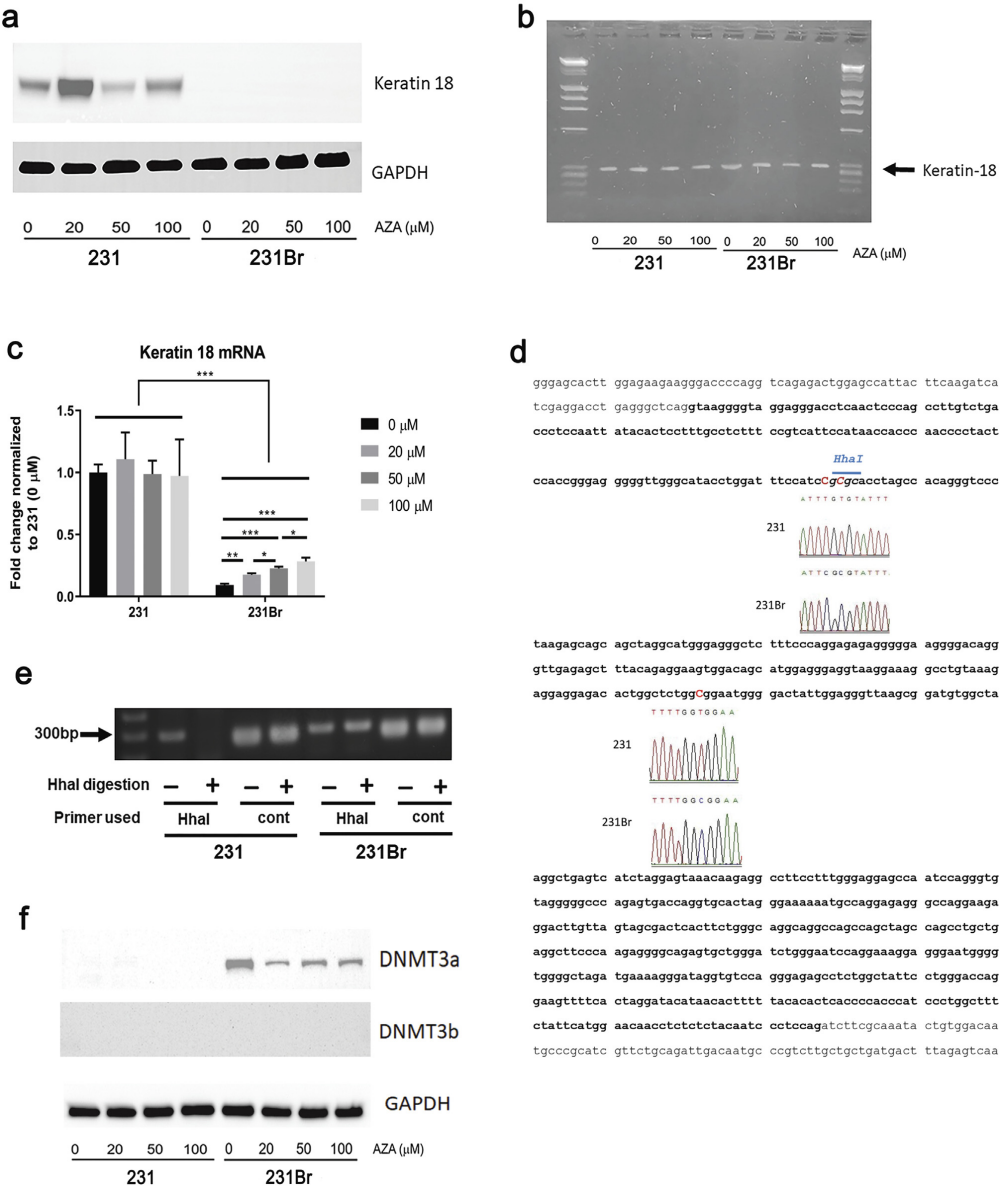
The keratin 18 gene is hypermethylated in brain colonizing cells compared to regular breast cancer cells. (A) Expression of keratin 18 in 231 and 231Br cells after AZA treatment for 72 hours measured by Western blotting assay. GAPDH was used as the loading control. The blots shown are a presentation of two independent experiments. (B) Detection of the keratin 18 gene in 231 and 231Br cells after AZA treatment for 72 hours by PCR. The image shown is a presentation of two independent experiments. (C) Detection of the mRNA level of keratin 18 gene in 231 and 231Br cells after AZA treatment for 72 hours by real-time PCR. All error bars represent SD, *N* = 3 technical replicates, representative of two independent experiments. *P < .05, **P < .01, ***P < .001. (D) Detection and comparison of DNA methylation in the intron 1 region of keratin 18 gene between 231 and 231Br cells. Bold letters indicate the intron 1 region of keratin 8 gene. Inserts show the sequencing chromatogram of bisulfide-converted DNA from 231 and 231Br cell lines. Sequence in italics shows the HhaI restriction enzyme target site. The DNA sequencing results represents sequencing five positive colonies generated from each pair of primers. (E) Digestion of the genomic DNA isolated from 231 or 231Br cells with HhaI restriction enzyme. HhaI primer stands for the use of the pair of primers to detect the HhaI digestion by PCR (yielding a ~300-bp PCR product if the DNA was not digested by HhaI; no such ~300-bp PCR product was formed if the DNA was digested by HhaI). Control primer (“cont” in figure) is the pair of primers used as positive control to detect the keratin 18 gene by PCR. The image shown is a presentation of two independent experiments. (F) Expression of DNMT3a and DNMT3b in 231 and 231Br cells after AZA treatment for 72 hours measured by Western blotting assay. GAPDH was used as the loading control. The blots shown are a presentation of two independent experiments.

Cytokeratin 18 is an epithelial cytokeratin coded by the keratin 18 (or KRT18) gene [28]. We detected expression of keratin 18 protein in 231 cells but not in 231Br cells by Western blotting assay (Figure 5*A*), despite the keratin-18 gene being present in both cell linesas detected by PCR (Figure 5*B*). Also, it should be noted that the mRNA levels of the keratin gene 18 were significantly lower in the 231Br cells (*P* < .001, Figure 5*C*). We noted that AZA treatment increased keratin 18KRT18 mRNA significantly in a dose-dependent manner in 231Br cells (every dose increased with significance at *P* < .05 or lower, Figure 5*C*). Based on these data, we hypothesized that decreased gene expression may be due to DNA hypermethylation. It has been shown that DNA methylation of intron 1 in the keratin 18 gene is important in regulating expression [29]. We did not detect DNA mutation or deletion of this region (737 bp) of the keratin 18 gene in either cell line, and the DNA sequence is identical (data not shown). We sequenced and compared DNA methylation of the intron 1 region in the two cell lines by using the bisulfite conversion method. We observed that three cytosine residues were converted into uracil in 231 cells, while they remained unchanged in 231Br cells (Figure 5*D*). Among the three identified different cytosine sites, one is the target of the HhaI restriction enzyme (Figure 5*D*). To further confirm the difference in particular cytosine methylation, the genomic DNA from both cell lines was digested with HhaI following PCR using designed primers. A ~300-bp band was detected in 231Br cells but not in 231 cells, confirming cytosine methylation of keratin gene (Figure 5*E*). DNA methylation is catalyzed by DNA methyltransferases (DNMTs) which include two major types, DNMT3a and DNMT3b, and accordingly, we measured the expression of the enzymes with AZA treatment [6]. The expression of DNMT3a was only detected in 231Br cells, and the expression of DNMT3b was undetectable in either cell type (Figure 5*F*). AZA treatment decreases the DNMT3a expression in 231Br cells, indicating that AZA can potentially inhibit the process of DNA methylation in brain colonizing cells (Figure 5*F* and Supplementary Table 2).

### AZA Decreased Tumor Burden and Prolonged Survival in Mice with Brain Metastases of Breast Cancer

**Figure 6:**
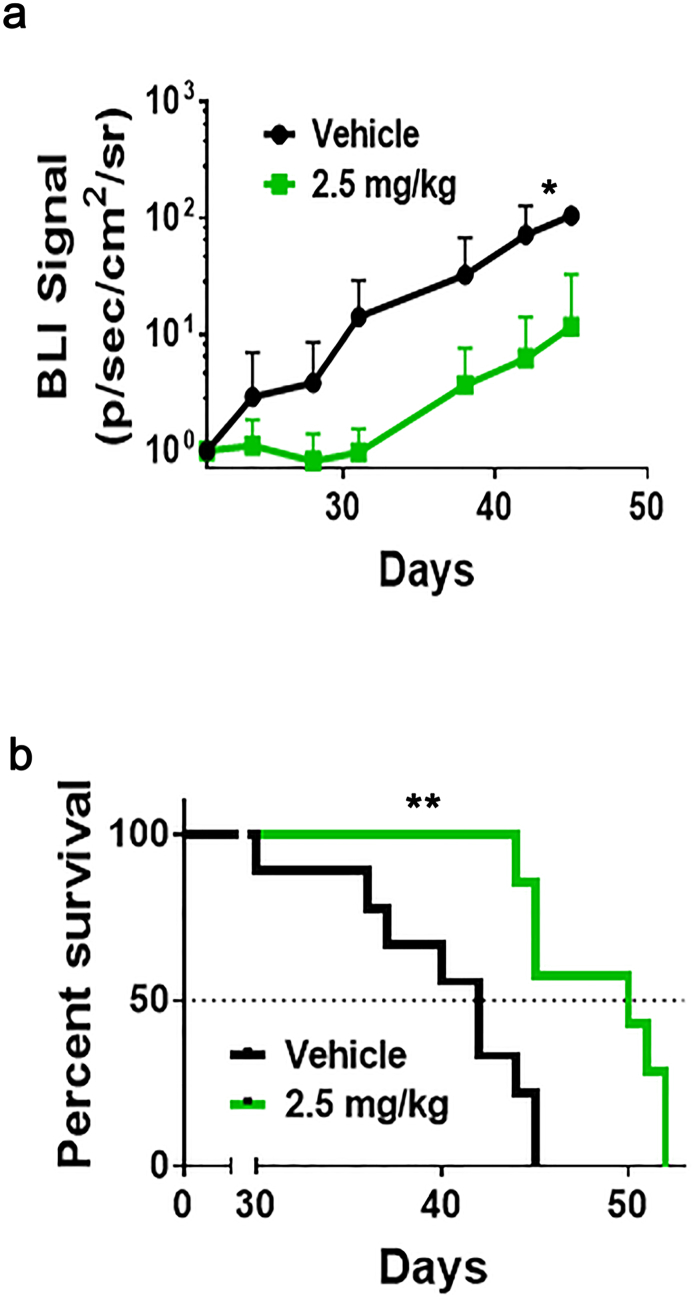
AZA decreases tumor burden and improves survival in mice with brain metastasis of breast cancer. (A) BLI signal versus time in mice with AZA or PBS treatment beginning on day 21 after intracardiac injection with 231Br cells. Each data point represents the mean plus SD. Mice treated with 2.5 mg/kg AZA had significantly lower tumor burden (*P* = .0112). (B) Kaplan-Meier survival plot of mice starting 21 days after intracardiac injection of 231Br cells. Median survival was 42 days and 50 days, respectively, for vehicle and 2.5 mg/kg AZA (*P* = .0026).

To determine if AZA treatment improved survival and control of tumor burden *in vivo*, we injected mice intracardially with the 231Br cells and allowed for development of metastatic brain lesions [22,23]. After 21 days, tumor-bearing mice were randomized into vehicle (PBS) and drug treatment (AZA, 2.5 mg/kg body weight) groups. We observed that, in mice treated with 2.5 mg/kg AZA, tumor burden was significantly lower compared to vehicle-treated mice (*P* = .0112, Figure 6*A*). We also noted that AZA treatment significantly increased survival when compared to vehicle-treated mice, with median survival of 50 and 42 days, respectively (*P* = .0026, Figure 6*B*).

## Discussion

DNA methylation is an epigenetic mechanism used by cells to control gene expression [6]. DNA hypermethylation may cause improper gene silencing, leading to the downregulation of gene expression and alleviation of gene function. Hypermethylation of numerous tumor suppressor genes has been identified in multiple cancer types, suggesting that DNA hypermethylation may contribute to the initiation, development, and increased metastatic capacity of cancer [7,10].

The hypomethylating agent AZA and its deoxyl derivative 2′-deoxy-5′-azacytidine (decitabine) were developed as pyrimidine nucleoside analogs in 1960s. Later, it was observed that the compounds inhibit DNA methylation in human cell lines [30]. Studies of AZA also showed antitumor activity in hematological malignancies including MDS, acute myeloid, chronic myeloid, and acute lymphocytic leukemia [31]. Concurrent epigenetic work showed that multiple important genes are hypermethylated in MDS patients. One of the genes is tumor suppressor gene CDKN2B, which encodes the cyclin-dependent kinase inhibitor p15^INK4b^. Other hypermethlated genes in MDS patients include the calcitonin gene, HIC, E-cadherin, and estrogen receptor [32]. The led to the FDA approval of AZA as the first therapy for all subtypes of MDS in 2004 [33]. With relevance to this work, in breast cancer, multiple genes (e.g., p16, p53, and BRAC1) are also hypermethylated [[12], [13], [14]]. Breast cancer brain metastasis poses a life-threatening problem for women with advanced metastatic breast cancer, and current chemotherapeutic agents are largely ineffective against brain metastases [[34], [35], [36], [37], [38]]. In this study, we tested the effectiveness of the hypomethylating agent AZA in treating brain metastasis of breast cancer using a combined *in vitro* cell and *in vivo* approach [22,23].

We observed that the IC_50_ value of AZA in 231Br cells was significantly lower than in 231Br cells (Figure 2*A*) and that AZA treatment triggered a higher percentage of apoptosis in 231Br cells compared to 231 cells (Figure 2, *B*-*D* and Supplementary Table 2). Further, AZA inhibited BCL-2 expression in 231Br cells in a dose-dependent manner, suggesting that inhibition of antiapoptotic BCL-2 may be a mechanism of antitumor therapeutic response induced by AZA in these cells. Overall, these results suggest that 231Br cells are more sensitive to AZA treatment. In triple-negative breast cancer, Wnt signaling regulates cell differentiation, proliferation, and stem cell pluripotency [39,40]. Accordingly, we examined AZA effects in Wnt signaling and observed that expression of Wnt-3, Wnt-4, GSK-3, and beta-catenin was inhibited by AZA in a dose-dependent manner in 231Br cells (Figure 3*A* and Supplementary Table 2). As beta-catenin is required for the tumorigenic behavior of triple-negative cancer cells, our results suggest that AZA inhibits Wnt signaling as well as tumorigenesis in brain colonizing cells more [41]. Consistent with previous literature, we did not observe differences in the Ras/Raf/MEK/MAPK and PI3K/Akt/mTOR pathways as they have greater influence in HER2+ cancer (Supplementary Figure 2, *B* and *C*) [42]. We also observed that AZA treatment inhibiteds (Figure 3, B-D, and Supplemental Table 2), cell migration, and cell invasion (Figure 4) more dramatically in brain colonizing cells. Collectively, *in vitro* results support the hypothesis that AZA is effective in treating brain metastasis of breast cancer *in vivo*.

The BBB acts as a physiological and biochemical barrier that restricts the passage of many hydrophilic and large–molecular weight compounds. AZA is a nucleic acid synthesis inhibitor with a molecular weight of 244.2 g/mol and an XLogP3 of −1.9. Strictly based on its physicochemical properties, AZA serves as a model compound to cross biological membranes like the BBB with an ideal range of molecular weight, lipophilicity, and hydrogen bond donors and acceptors. Additionally, its relatively high aqueous solubility and stability render it as a potentially advantageous investigative chemotherapeutic for brain delivery [[43], [44], [45], [46]]. Since AZA is able to cross the BBB [16,17], we next used an *in vivo* mouse model of brain metastasis of breast cancer and treated animals with AZA (2.5 mg/kg body weight) or vehicle control (PBS). Our *in vivo* studies showed that the overall survival of AZA-treated mice was significantly increased compared to mice treated with PBS (Figure 6*A*). The *in vivo* BLI assay also suggested that AZA significantly inhibited the tumor activity in mice compared to PBS (Figure 6*B*).

Decitabine has been shown to reverse gefitinib resistance caused by DAPK gene promoter methylation in lung cancer cells, suggesting a role of DNA methylation in drug resistance and cancer progression [47]. Thus, after confirming the effectiveness of AZA in treating brain metastasis triple-negative breast cancer *in vitro* and *in vivo*, we explored the molecular mechanism of action of AZA. EMT is defined by the loss of epithelial and acquisition of mesenchymal characteristics, which promote cancer cell progression, invasion, and metastasis into surrounding microenvironment [48,49]. Cytokeratins are major structural proteins found in epithelial cells, forming the cytoplasmic network of intermediate filaments [50]. As important epithelial makers, the expression of cytokeratins is decreased during the EMT process, which may contribute to breast cancer metastasis [27]. The human cytokeratin family consists of at least 20 members coded by different cytokeratin genes including the keratin 18 gene, which is located on chromosome 12q13 with 3791 bp [28,51]. Keratin 18 plays biological functions in carcinogenesis, and its expression may serve as a differential diagnostic marker in various cancers such as small cell lung cancer and breast cancer [[52], [53], [54]]. In order to explore role of keratin 18 gene in breast cancer brain metastasis and hypomethylating agent treatment, we first measured the expression of the keratin 18 DNA, mRNA, and protein. We observed that the keratin 18 gene was present in both cell lines (Figure 5*B*), but its transcription and translation were dramatically decreased in 231Br cells (Figure 5, *C* and *A*). Moreover, AZA treatment increased the mRNA level of keratin 18 in a dose-dependent manner (Figure 5*C*). Previous studies have shown that the first intron of the keratin 18 gene contains GC-rich regions with DNA methylation sites, which are important in regulating its expression [29,55]. Thus, we hypothesized that the decreased keratin 18 gene expression in 231Br cells is due to DNA hypermethylation. We sequenced and compared the DNA sequence of intron 1 (737 bp) of the keratin 18 gene between both cell lines, and we found that the DNA sequence was identical, further indicating that decreased expression of keratin 18 in 231Br cells may due to DNA hypermethylation. Further, we identified three cytosines that were converted into uracil in 231 cells, suggesting that DNA methylation status of keratin 18 was different between both cell lines (Figure 5*D*). Further HhaI restriction enzyme digestion following PCR analysis confirmed that at least one of the three cytosine sites is methylated in 231Br but not in 231 cells (Figure 5*E*). These results strongly suggest that the keratin 18 gene is hypermethylated in 231Br cells.

So far, two mechanisms of action of hypomethylating agents have been reported: 1) incorporation of drugs into DNA strands and prevention of RNA synthesis and 2) inhibition of the activity of DNMTs which catalyze the process of DNA methylation [6,7,11]. Targeting DNA methylation may be the general mechanism of these agents; however, the precise mechanism of action of hypomethylating agents in cancer treatment has not been elucidated. DNMTs are enzymes that catalyze the addition of methyl groups to cytosine residues in DNA. DNMTs found in mammalian cells include DNMT1, DNMT3a, DNMT3b. DNMT1 and DNMT3b are found to plays roles in the development of central nervous system, while DNMT3a has important functions in acute myeloid leukemia [[56], [57], [58], [59]]. We measured the expression of DNMT3a and DNMT3b and found the expression of DNMT3a is only present in 231Br cells, while DNMT3b expression is undetectable in either cell line. We also noticed that AZA inhibited the DNMT3a expression in 231Br cells (Figure 5*F* and Supplementary Table 2). The presence of DNMT3a and inhibition of its expression by AZA in the brain colonizing cells suggest that 1) DNA methylation is elevated and 2) AZA inhibits DNA methylation in these cells. Comparing different methylation status of the keratin 18 gene between the two cell lines, the difference in DNMT3a expression provides a correlation and explanation of DNA methylation, brain metastasis, and effectiveness of AZA in brain colonizing cells.

In summary, based upon our findings, we believe that the DNA hypomethylating agent AZA may represent a new class of chemotherapeutic agents and a novel therapy for treatment of brain metastasis of breast cancer. A recent study showed that epigenetically reprogrammed genomic methylation serves as a universal cancer biomarker [58]. We propose that DNA hypermethylation of the keratin 18 gene may serve as a biomarker for diagnosis of brain metastasis of breast cancer or can be used to evaluate whether breast cancer patients with brain metastasis are potential candidates and would benefit from hypomethylating agent treatment. Moreover, the hypermethylated keratin 18 gene may be a potential drug target that can be used for the development of novel targeted therapy drugs in treating patients with brain metastasis breast cancer.

## Appendix A Supplementary data

Supplementary material Image 1
