# Supplementary material for: Hypomethylating Agent Azacitidine Is Effective in Treating Brain Metastasis Triple-Negative Breast Cancer Through Regulation of DNA Methylation of Keratin 18 Gene
Source: Transl Oncol. 2020 May 11;13(6):100775. doi: 10.1016/j.tranon.2020.100775 (PMC7225776; doi:10.1016/j.tranon.2020.100775)
Supplement: Supplementary file 1 — Supplementary material [file mmc1.pdf]

Supp. Fig. 1

a

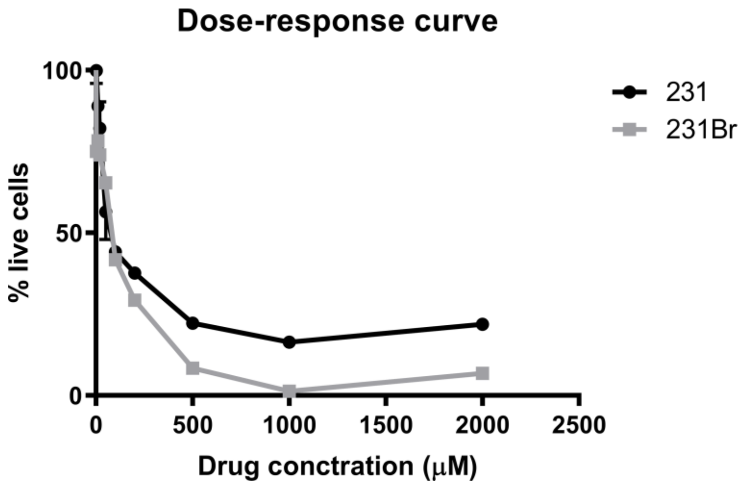
Dose-response curve
100
50
0
% live cells
0
500
1000
1500
2000
2500
Drug conctration (μM)
231
231Br

b

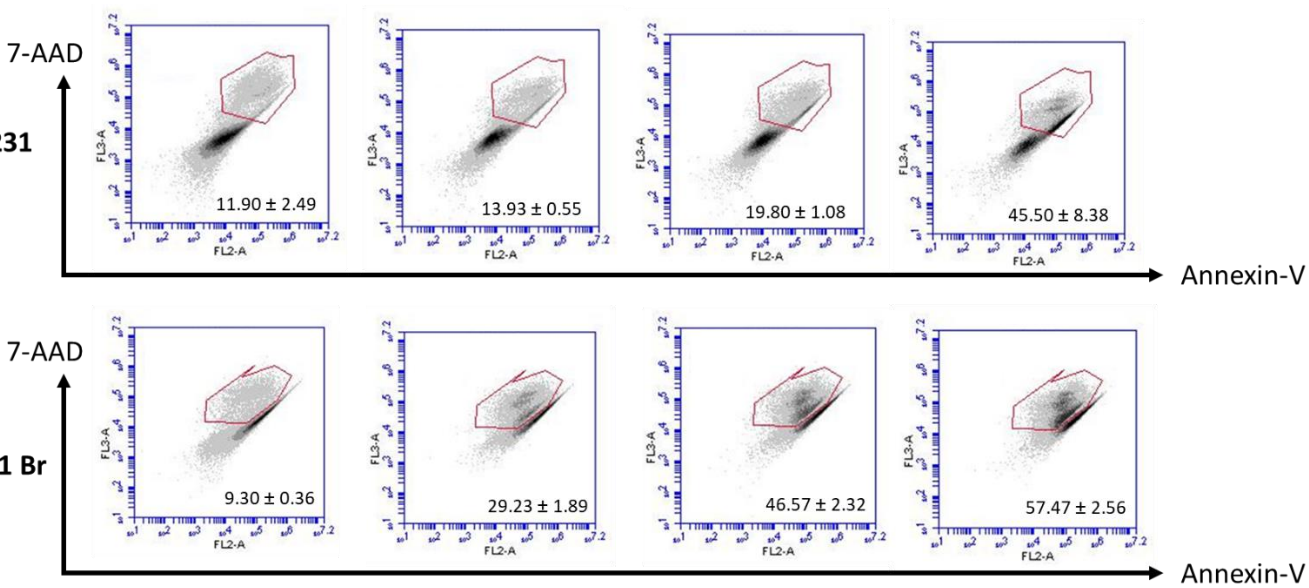
7-AAD
231
11.90 ± 2.49
13.93 ± 0.55
19.80 ± 1.08
45.50 ± 8.38
Annexin-V
7-AAD
231 Br
9.30 ± 0.36
29.23 ± 1.89
46.57 ± 2.32
57.47 ± 2.56
Annexin-V
AZA conc. 0 μM
20 μM
100 μM
500 μM

c

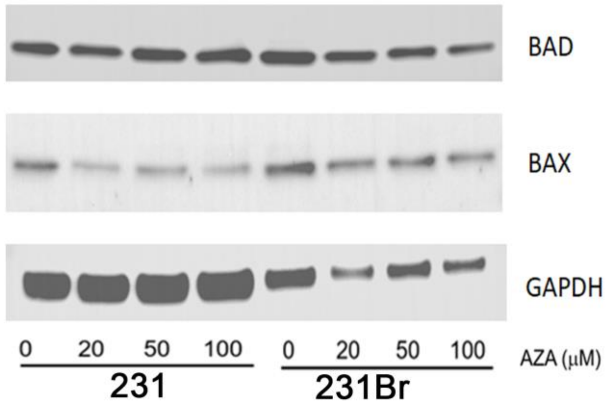

Supp. Fig. 1. Brain metastasized breast cancer cells (231Br) have different response to AZA treatment compared to their parental regular breast cancer 231 cells. (a) Dose-response curve used to calculate IC 50 values of AZA in 231 and 231Br cells ($p<0.01$). The figure represents N = 3 technical replicates, three independent experiments. (b) Flow cytometry plot for the detection of percentage of Annexin-V positive cells after various concentrations of AZA treatment for 3 days. The Annexin-V positive cells were gated based on unstained negative control and Annexin-V only stain positive control cells. The plot represents N = 3 technical replicates, two independent experiments. (b) Expression of BAD and BAX in 231 and 231Br cells after AZA treatment for 3 days measured by Western blotting assay. GAPDH was used as the loading control. The blots shown are a presentation of two independent experiments.

Supp. Fig. 2

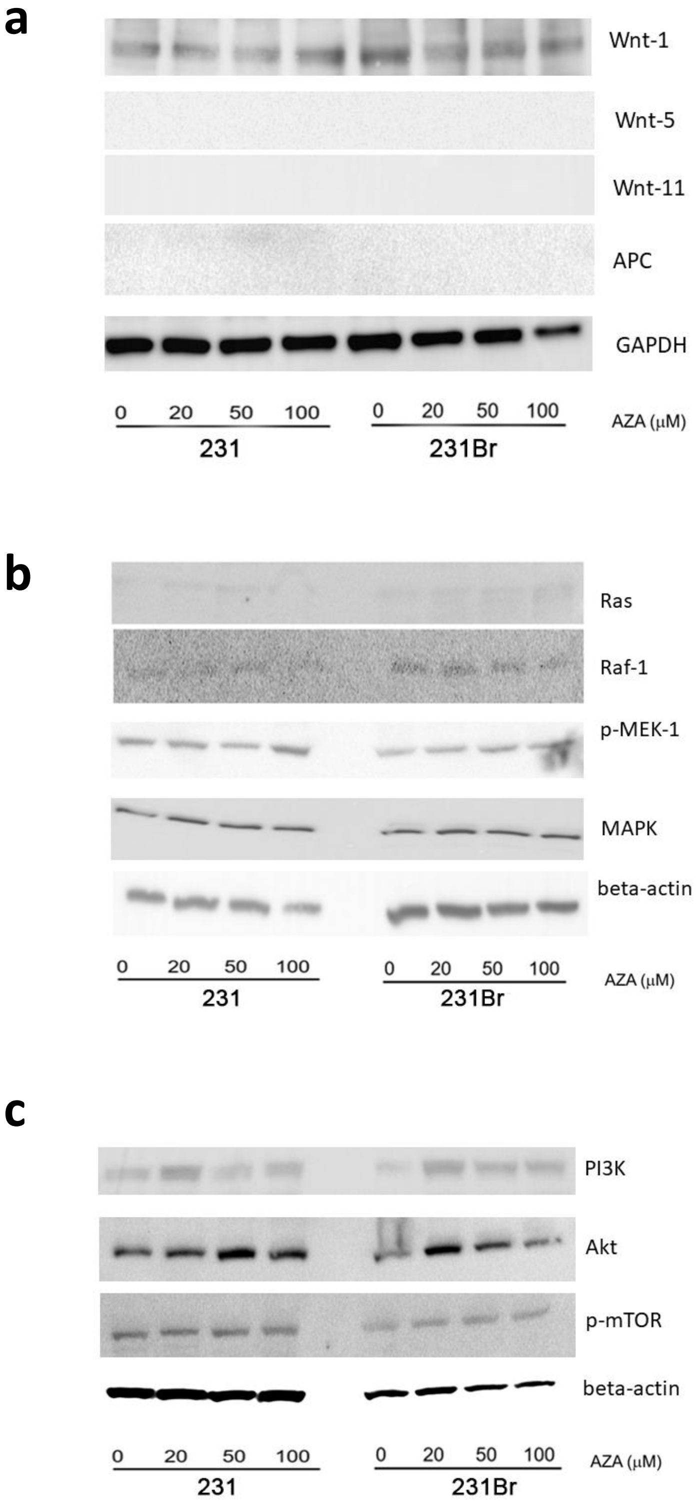
a
Wnt-1
Wnt-5
Wnt-11
APC
GAPDH
0 20 50 100
0 20 50 100
AZA (μM)
231
231Br
b
Ras
Raf-1
p-MEK-1
MAPK
beta-actin
0 20 50 100
0 20 50 100
AZA (μM)
231
231Br
c
PI3K
Akt
p-mTOR
beta-actin
0 20 50 100
0 20 50 100
AZA (μM)
231
231Br

Supp. Fig. 2. Inhibition of Wnt, Ras-Raf-MEK-MAPK, PI3K-Akt-mTOR signaling transduction pathways by AZA in two cells lines. (a) Expression of Wnt-1 Wnt-5, Wnt-11, and APC in 231 and 231Br cells after AZA treatment for 3 days measured by Western blotting assay. GAPDH was used as the loading control. The blots shown are a presentation of two independent experiments. (b) Expression of Ras, Raf-1, phosphorylated MEK-1, and MAPK in 231 and 231Br cells after AZA treatment for 3 days measured by Western blotting assay. Beta-actin was used as the loading control. The blots shown are a presentation of two independent experiments. (c). Expression of PI3K, Akt, and phosphorylated mTOR in 231 and 231Br cells after AZA treatment for 3 days measured by Western blotting assay. Beta-actin was used as the loading control. The blots shown are a presentation of two independent experiments.

Supp. Fig. 3

a

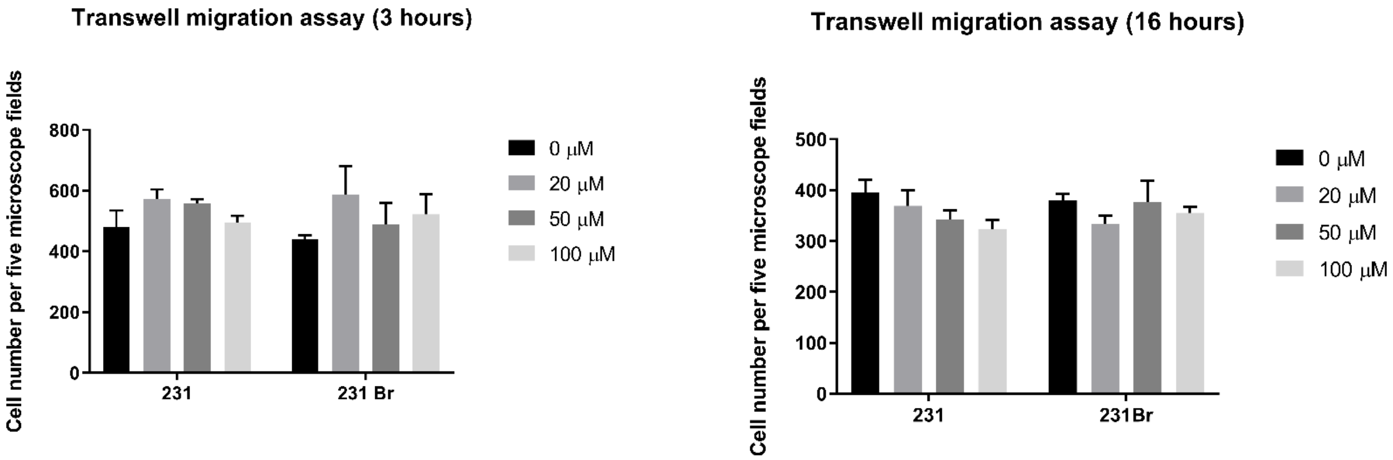
Transwell migration assay (3 hours)
Cell number per five microscope fields
800
600
400
200
0
231
231 Br
0 μM
20 μM
50 μM
100 μM
Transwell migration assay (16 hours)
Cell number per five microscope fields
500
400
300
200
100
0
231
231Br
0 μM
20 μM
50 μM
100 μM

b

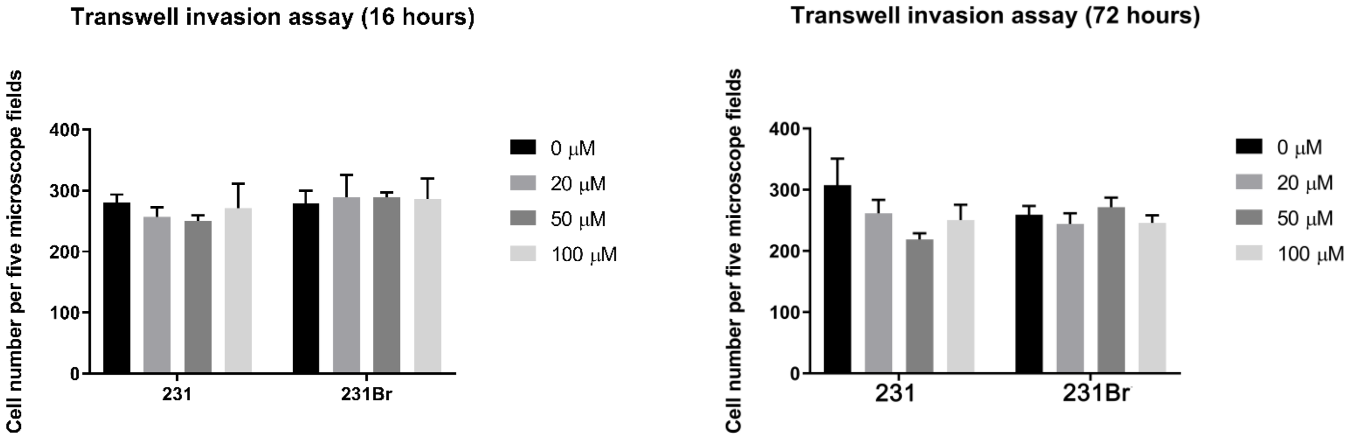
Transwell invasion assay (16 hours)
Cell number per five microscope fields
400
300
200
100
0
231
231Br
0 μM
20 μM
50 μM
100 μM
Transwell invasion assay (72 hours)
Cell number per five microscope fields
400
300
200
100
0
231
231Br
0 μM
20 μM
50 μM
100 μM

c

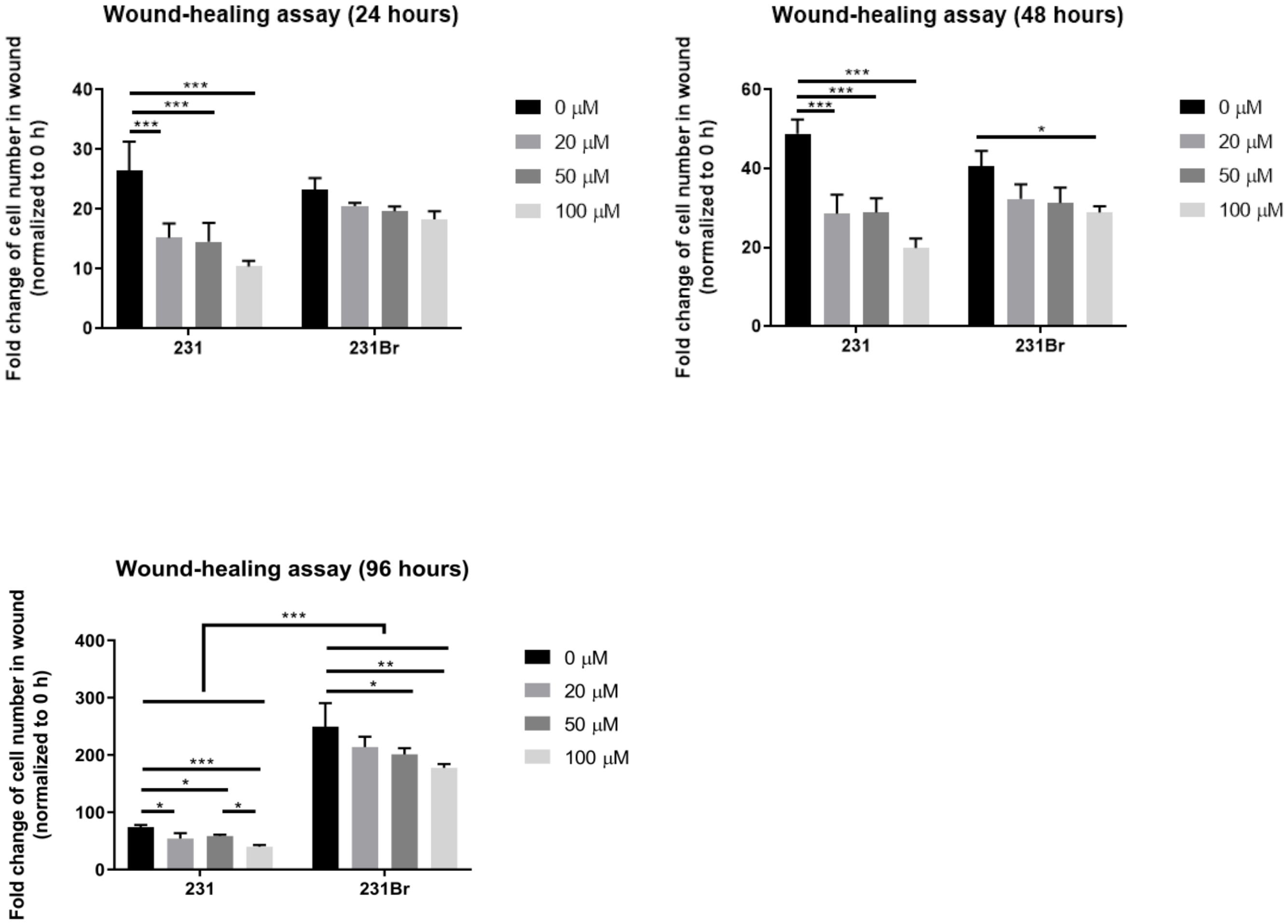
Wound-healing assay (24 hours)
Fold change of cell number in wound (normalized to 0 h)
0
10
20
30
40
***
231
231Br
0 μM
20 μM
50 μM
100 μM
Wound-healing assay (48 hours)
Fold change of cell number in wound (normalized to 0 h)
0
20
40
60
***
*
231
231Br
0 μM
20 μM
50 μM
100 μM
Wound-healing assay (96 hours)
Fold change of cell number in wound (normalized to 0 h)
0
100
200
300
400
***
**
*
231
231Br
0 μM
20 μM
50 μM
100 μM

d

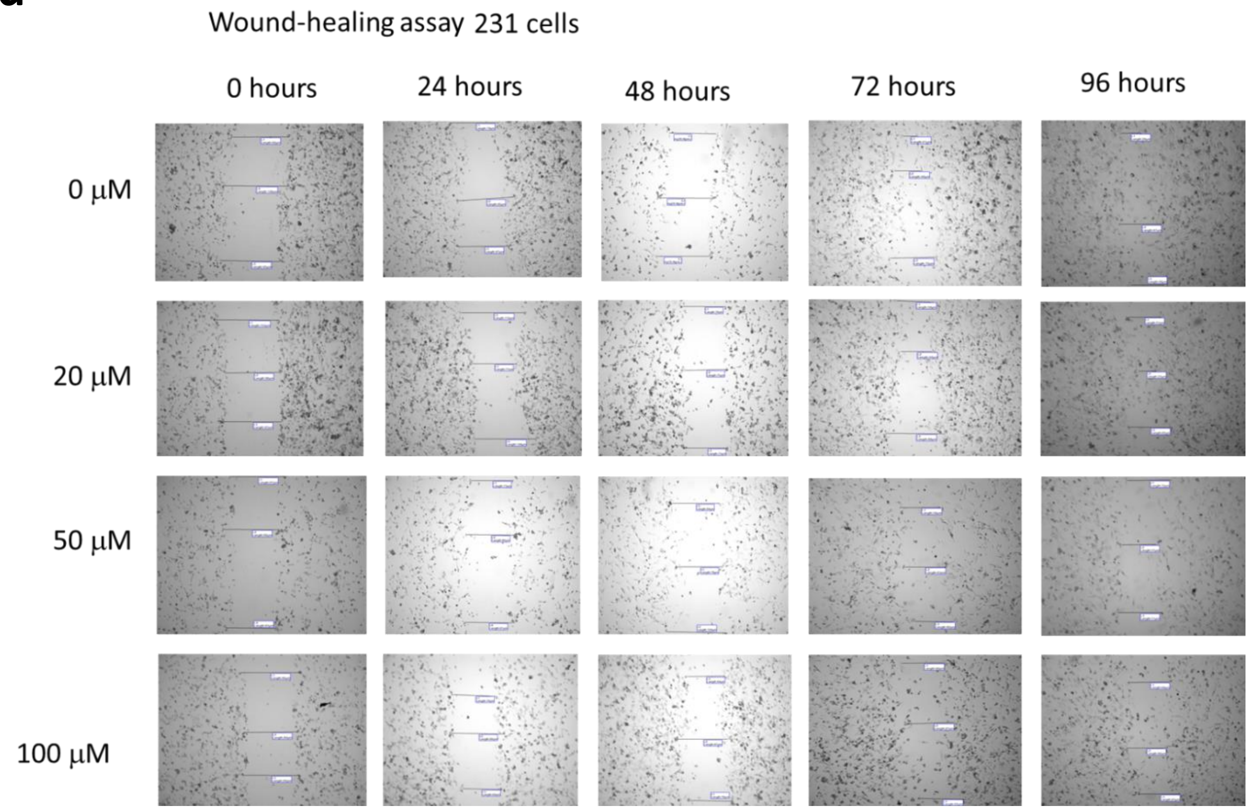
Wound-healing assay 231 cells
0 hours
24 hours
48 hours
72 hours
96 hours
0 μM
20 μM
50 μM
100 μM

e

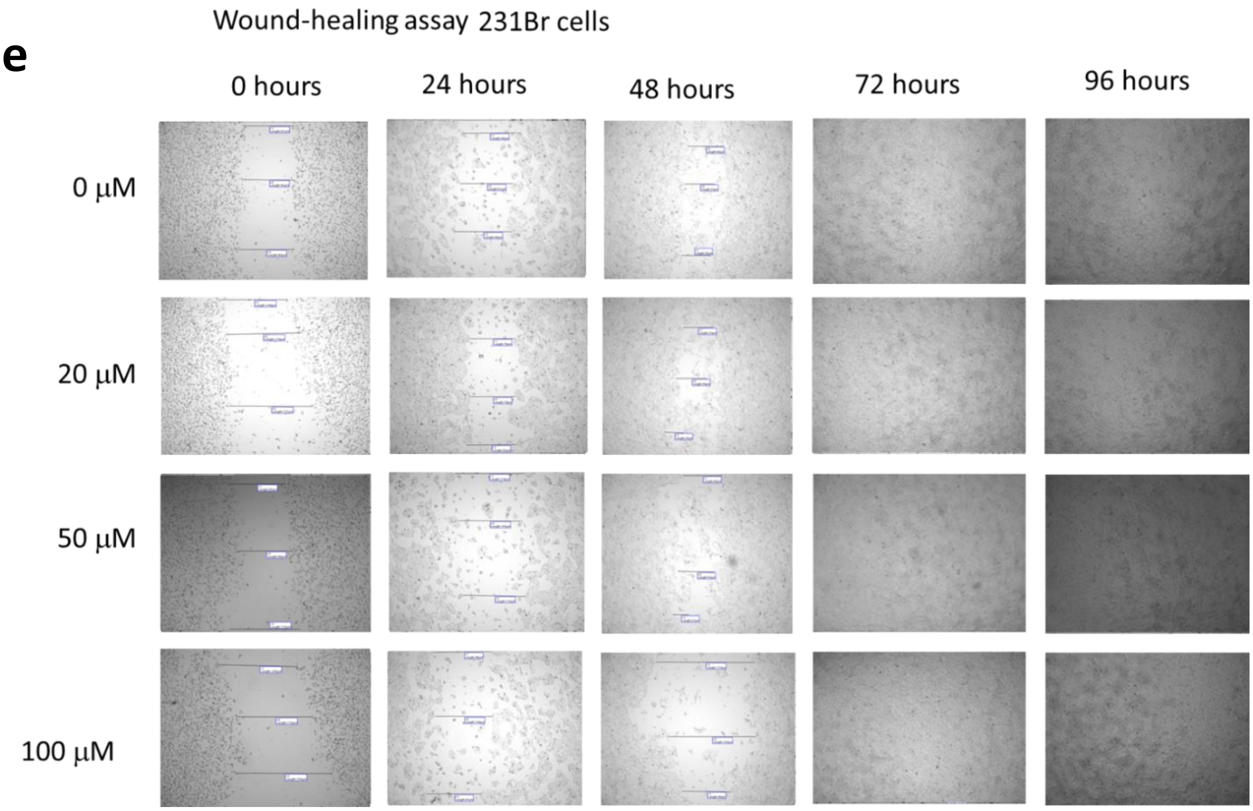
Wound-healing assay 231Br cells
0 hours
24 hours
48 hours
72 hours
96 hours
0 μM
20 μM
50 μM
100 μM

Supp. Fig. 3. Brain colonizing cells have higher migration and invasion potential compared to regular breast cancer cells. (a) Quantification of cells migrating across transwells 3 and 16 hours after plating cells in the migration chambers measured by transwell migration assay. Y-axis stands for the average number of cell migration per 5 microscope fields. All error bars represent standard deviation (SD), representative of two independent experiments. (b) Quantification of cells migrating across transwells 16 and 72 hours after plating cells in the Matrigel coated migration chambers measured by transwell invasion assay. Y-axis stands for the average number of cell migration per 5 microscope fields. All error bars represent standard deviation (SD), representative of two independent experiments. (c) Quantification of cells presented in the scratch made on day 0 (0 hour time point) at 24, 48, and 96 hours after AZA treatment by wound-healing assay. Cell numbers in the scratch wound were normalized to 0 hour. All error bars represent standard deviation (SD), representative of two independent experiments. *$p<0.05$, **$p<0.01$, ***$p<0.001$. (d) Width of the scratch wound made on day 0 measured at 0, 24, 48, 72 and 96 hours in 231 and 231Br cells with AZA treatment. The images represent N = 3 technical replicates, two independent experiments. (e) Representative microscopic images of the wound area migrated by 231 cells treated by different concertation of AZA at different time points. The width of the wound was measured and labelled with scale. (f) Representative microscopic images of the wound area migrated by 231Br cells treated by different concertation of AZA at different time points. The width of the wound was measured and labelled with scale.

Supp. Fig. 4

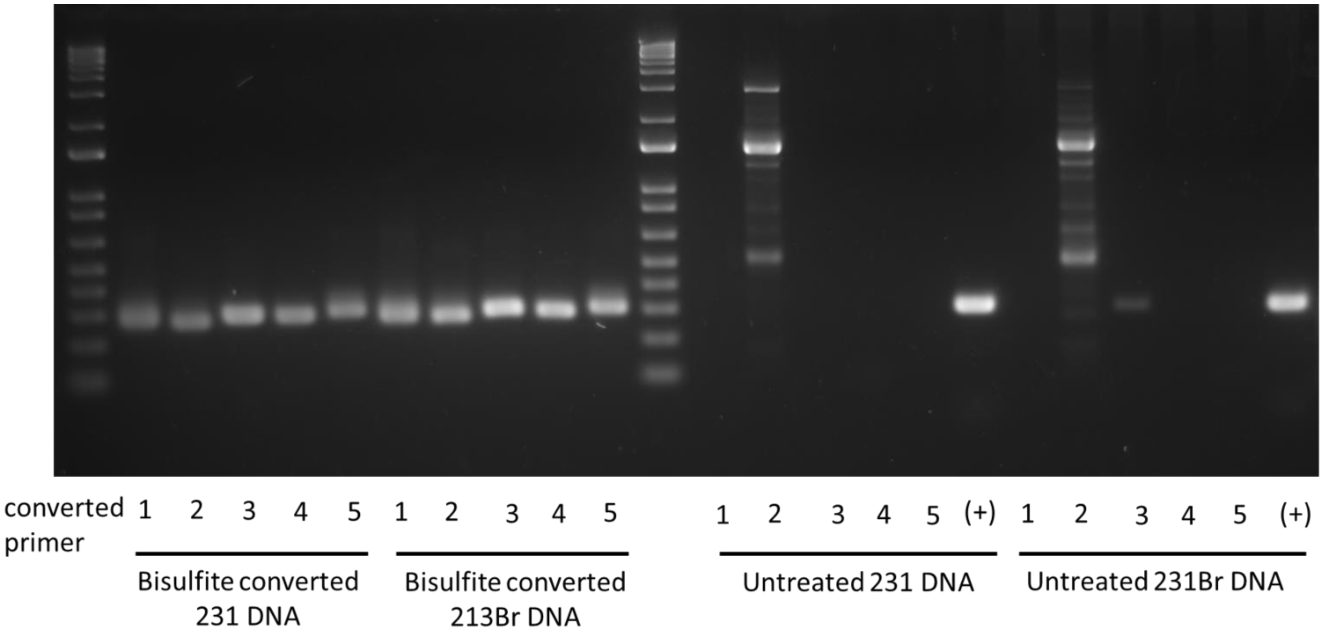

Supp. Fig. 4. Five pairs of primers were designed and used to amplify the bisulfite modified intron 1 region of keratin 18 gene by PCR. In order to measure and compare the DNA methylation of the keratin 18 gene in both cell lines, five pairs of primers (converted primer 1-5) were designed and used to fully cover and amplify the bisulfite modified intron 1 region of keratin 18 gene by PCR. Each of the five pairs of primers yielded a single and clear PCR band using bisulfite converted genomic DNA as the template from both MDA-MB-231 and MDA-MB-231 Br cell lines. Each of the five pairs of primers could not yielded a single and clear PCR band using regular (non-bisulfite converted) genomic DNA as the template from both cell lines.

Supp. Table 1. Information of the antibodies used in Western blotting assays

Antibody name (clone)	Manufacture	Catalog number
Bcl-2 (C-2)	Santa Cruz biotechnology	sc7382
Bcl-xL (H-5)	Santa Cruz biotechnology	sc8392
Caspase-3 (E-8)	Santa Cruz biotechnology	sc7272
Caspase-9 p35 (A-9)	Santa Cruz biotechnology	sc133109
beta actin (AC-15)	Sigma-Aldrich	A5441
Bad (C-7)	Santa Cruz biotechnology	sc8044
Bax (6A7)	Santa Cruz biotechnology	sc23959
GAPDH (14C10)	Cell signaling	14C10
Wnt-1 (E-10)	Santa Cruz biotechnology	sc514531
Wnt-3 (3A6)	Santa Cruz biotechnology	sc136163
Wnt-4 (B-6)	Santa Cruz biotechnology	sc376279
Wnt-5 (A-5)	Santa Cruz biotechnology	sc365370
Wnt-11 (C-8)	Santa Cruz biotechnology	sc365032
APC (F-3)	Santa Cruz biotechnology	sc9998
GSK-3alpha/beta (0011-A)	Santa Cruz biotechnology	sc7291
Phosphorylated GSK-3	Cell signaling	9331
beta catenin (E-5)	Santa Cruz biotechnology	sc7963
Phospho-beta-catenin	Invitrogen	PA5-17915
pan-Ras (F132)	Santa Cruz biotechnology	sc32
Raf-1 (E-10)	Santa Cruz biotechnology	sc7267
p-MEK-1 (47.Ser 222)	Santa Cruz biotechnology	sc136542
ERK 1 (G-8) / MAPK	Santa Cruz biotechnology	sc271269
PI 3-kinase p85alpha (C1)	Santa Cruz biotechnology	sc376112
Akt1 (B-1)	Santa Cruz biotechnology	sc5298
p-mTOR (59.Ser 2448)	Santa Cruz biotechnology	sc293133
HIF-1alpha (H-206)	Santa Cruz biotechnology	sc10790
VEGF (C-1)	Santa Cruz biotechnology	sc7269
VEGF receptor 1	R&D Systems	49560
VEGF receptor 2	R&D Systems	AF357
TGF beta (3C11)	Santa Cruz biotechnology	sc130348
MMP-2	ABCam	ab37150
MMP-7	ABCam	ab5706
MMP-9 (2C3)	Santa Cruz biotechnology	sc21733
Vimentin (V9)	Santa Cruz biotechnology	sc6260
N-cadherin (13A9)	Santa Cruz biotechnology	sc59987
E-cadherin (G-10)	Santa Cruz biotechnology	sc8426
pan-Cytokeratin (AE1/AE3)	Santa Cruz biotechnology	sc81714
Keratin 18 (E431-1)	ABCam	ab32118
DNMT3a (C-12)	Santa Cruz biotechnology	sc365769
DNMT3b (G-9)	Santa Cruz biotechnology	sc376043

Supp. Table 2. Quantification of Western blotting images by densitometry

Fig. 2c.

	231				231Br			
**AZA conc.(μM)**	**0**	**20**	**50**	**100**	**0**	**20**	**50**	**100**
**BCL-2/beta-actin**	0.43	0.61	0.83	0.63	0.69	0.46	0.42	0.31
**Normalized to 0 μM AZA**	1	1.40	1.93	1.46	1	0.67	0.61	0.44

Fig. 2d.

	231				231Br			
**AZA conc.(μM)**	**0**	**20**	**50**	**100**	**0**	**20**	**50**	**100**
**Procaspase 9 (upper band)/beta-actin**	0.63	0.89	0.68	0.79	0.94	1.03	1.11	1.92
**Normalized to 0 μM AZA**	1	1.40	1.08	1.24	1	1.10	1.17	2.04
**Cleaved caspase 9 (lower band)/beta-actin**	1.02	1.12	0.93	2.31	0.91	1.26	1.28	1.38
**Normalized to 0 μM AZA**	1	1.09	0.91	2.25	1	1.38	1.41	1.51
**AZA conc.(μM)**	**0**	**20**	**50**	**100**	**0**	**20**	**50**	**100**
**Caspase 3 /beta-actin**	1.14	1.24	1.32	1.25	0.72	0.73	0.97	1.07
**Normalized to 0 μM AZA**	1	1.08	1.15	1.10	1	1.01	1.35	1.48

Fig. 3a.

	231				231Br			
**AZA conc.(μM)**	**0**	**20**	**50**	**100**	**0**	**20**	**50**	**100**
**Wnt-3/ GAPDH**	1.11	0.65	0.71	0.56	0.83	0.85	0.24	0.22
**Normalized to 0 μM AZA**	1	0.58	0.64	0.50	1	1.02	0.29	0.25
**Wnt-4/ GAPDH**	0.28	0.68	0.67	0.066	1.56	1.06	1.05	0.18
**Normalized to 0 μM AZA**	1	2.43	2.39	0.23	1	0.68	0.68	0.11
**GSK-3 (upper band)/ GAPDH**	0.52	0.64	0.55	0.23	1.11	0.99	0.86	0.23
**Normalized to 0 μM AZA**	1	1.21	1.04	0.43	1	0.89	0.78	0.21
**GSK-3 (lower band)/ GAPDH**	0.66	0.68	0.58	0.21	1.09	0.85	0.56	0.27
**Normalized to 0 μM AZA**	1	1.02	0.87	0.32	1	0.78	0.51	0.24
**Beta-catenin/ GAPDH**	1.30	1.38	1.12	0.54	1.38	1.19	1.28	0.77
**Normalized to 0 μM AZA**	1	1.06	0.85	0.41	1	0.86	0.92	0.55

Fig. 3b.

	231				231Br			
**AZA conc.(μM)**	**0**	**20**	**50**	**100**	**0**	**20**	**50**	**100**
**VEGF receptor 2/GAPDH**	1.03	1.58	1.11	1.00	3.12	2.14	1.97	1.17
**Normalized to 0 μM AZA**	1	1.53	1.08	0.97	1	0.68	0.63	0.37
**HIF-1 alpha/GAPDH**	2.31	3.15	2.57	2.13	2.97	2.39	2.35	2.34
**Normalized to 0 μM AZA**	1	1.36	1.11	0.92	1	0.80	0.79	0.78

Fig. 4d.

	231				231Br			
**AZA conc.(μM)**	**0**	**20**	**50**	**100**	**0**	**20**	**50**	**100**
**MMP2/ GAPDH**	0.22	0.19	0.20	0.18	1.03	0.52	1.06	0.86
**Normalized to 0 μM AZA**	1	0.85	0.91	0.82	1	0.50	1.02	0.83
**MMP9/ GAPDH**					0.57	0.47	0.51	0.48
**Normalized to 0 μM AZA**					1	0.83	0.90	0.84
**Vimentin/ GAPDH**	1.03	1.09	0.86	0.87	0.75	0.86	0.86	0.72
**Normalized to 0 μM AZA**	1	1.05	0.82	0.84	1	1.14	1.14	0.95
**N-cadherin /GAPDH**					1.99	0.44	0.55	0.33
**Normalized to 0 μM AZA**					1	0.22	0.28	0.16

**Pan-cytokeratin/ GAPDH**	1.27	1.20	1.05	0.99	0.40	0.39	0.17	0.11
**Normalized to 0 μM AZA**	1	0.94	0.82	0.77	1	0.97	0.43	0.26

Fig. 5a.

	**231**				**231Br**			
**AZA conc.(μM)**	**0**	**20**	**50**	**100**	**0**	**20**	**50**	**100**
**Keratin 18/ GAPDH**	0.71	1.06	0.75	0.96				
**Normalized to 0 μM AZA**	1	1.48	1.06	1.35				

Fig. 5f.

	**231**				**231Br**			
**AZA conc.(μM)**	**0**	**20**	**50**	**100**	**0**	**20**	**50**	**100**
**DNMT3a/ GAPDH**					1.64	0.82	0.96	0.91
**Normalized to 0 μM AZA**					1	0.51	0.58	0.56
